# Immunoglobulin T from sea bass (*Dicentrarchus labrax* L.): molecular characterization, tissue localization and expression after nodavirus infection

**DOI:** 10.1186/s12867-017-0085-0

**Published:** 2017-03-15

**Authors:** Francesco Buonocore, Valentina Stocchi, Noelia Nunez-Ortiz, Elisa Randelli, Marco Gerdol, Alberto Pallavicini, Angelo Facchiano, Chiara Bernini, Laura Guerra, Giuseppe Scapigliati, Simona Picchietti

**Affiliations:** 1 Department for Innovation in Biological, Agro-food and Forest Systems, University of Tuscia, Largo dell’Università snc, 05100 Viterbo, VT Italy; 2 Department of Life Sciences, University of Trieste, Via Giorgieri 5, 34127 Trieste, TS Italy; 3 Institute of Food Science, CNR, Via Roma, 64, 83100 Avellino, AV Italy

**Keywords:** IgT, Sea bass, In situ hybridisation, Tissue expression, Mucosal immunity

## Abstract

**Background:**

Immunoglobulins (Igs) are fundamental components of the adaptive immune system of vertebrates, with the IgT/IgZ isotype specific of Teleosts. In this paper we describe the identification of an IgT heavy chain from the European sea bass (*Dicentrarchus labrax* L.), its molecular characterization and tissue mRNA localization by in situ hybridization.

**Results:**

Sea bass IgT consists of 552 aa (Accession Number KM410929) and it contains a putative 19 amino acids long signal peptide and one potential N-glycosylation site. The C-region consists of four C_H_ domains; each contains the cysteine and tryptophan residues required for their correct folding. Based on the recent sequencing of sea bass genome, we have identified five different genomic contigs bearing exons unequivocally pertaining to IgT (C_H_2, C_H_3 and C_H_4), but none corresponded to a complete IgH locus as IgT sequences were found in the highly fragmented assembled genomic regions which could not be assigned to any major scaffold. The 3D structure of sea bass IgT has been modelled using the crystal structure of a mouse Ig gamma as a template, thus showing that the amino acid sequence is suitable for the expected topology referred to an immunoglobulin-like architecture. The basal expression of sea bass IgT and IgM in different organs has been analysed: gut and gills, important mucosal organs, showed high IgT transcripts levels and this was the first indication of the possible involvement of sea bass IgT in mucosal immune responses. Moreover, sea bass IgT expression increased in gills and spleen after infection with nodavirus, highlighting the importance of IgT in sea bass immune responses. In situ hybridization confirmed the presence of IgT transcripts in the gut and it revealed a differential expression along the intestinal tract, with a major expression in the posterior intestine, suggesting the hindgut as a site for the recruitment of IgT^+^ cells in this species. IgT transcripts were also found in gill filaments and parallel lamellae and, for the first time, we identified scattered IgT positive cells in the liver, with a strong signal in the hepatic parenchyma.

**Conclusions:**

In conclusion, we performed a full molecular characterization of IgT in sea bass that points out its possible involvement in mucosal immune responses of this species.

## Background

Immunoglobulins (Igs) are critical factors of the adaptive immune system and they have been found in all vertebrates with jaws (gnathostomes) investigated to date [1]. Igs are composed of two heavy (H) and two light (L) chains and their repertoire is obtained through the recombination of variable (V), diversity (D) and joining (J) gene segments [2]. Different Igs isotypes have been identified in vertebrates, like: IgM, that is considered the most present within all species [3]; IgD, one of the less studied [4]; IgW, found in cartilaginous fish and in lungfish [5, 6]; IgY, found in amphibians, reptiles and birds [7]; IgG, IgE and IgA, found in mammals [1], and IgF, only found in amphibians [8].

In teleost fish only the presence of two Ig isotypes was recognized until about 10 years ago: IgM, a tetrameric molecule highly used to verify the specific immune-response against pathogens being the most abundant Ig in the serum [9], and IgD, a monomeric immunoglobulin whose function still needs to be fully characterized [10]. But in 2005 a new Ig was found at the same time in rainbow trout (*Onchorynchus mykiss*) and in zebrafish (*Danio rerio*) and named in two different ways: IgT (from trout, [11]) and IgZ (from zebrafish, [12]), respectively. After that, other IgT/IgZ sequences have been identified in different fish species such as in fugu (*Fugu rubripes*) [13], carp (*Cyprinus carpio*) [14], stickleback (*Gasterosteus aculeatus*) [15], Atlantic salmon (*Salmo salar*) [16, 17], Pacific bluefin tuna (*Thunnus orientalis*) [18] and, recently, in the emerald rockcod (*Trematomus bernacchii*) [19] and in the Atlantic salmon (*Salmo salar*) [20]. Only in catfish (*Ictalurus punctatus*) and medaka (*Oryzias latipes*), until now, appear to lack IgT orthologous genes [21, 22]. The functional characterization of IgT has been first performed in rainbow trout, where it has been considered mainly involved in mucosal immunity, thanks to the production of both polyclonal and monoclonal antibodies against this molecule [23]. The trout IgT was found in serum as a monomer, but not in gut mucus where it was identified as a tetramer with the different IgT monomers associated by non-covalent bonds [24, 25]. Moreover, IgT levels in gut mucus were double compared to serum IgT and even the ratio IgT/IgM was much higher in gut mucus compared to serum [23]. Another important finding was that IgT^+^ B cells could be considered as a new B cell lineage as they did not express either IgM or IgD transcripts [23]. Finally, with regard to the response against pathogens, IgT was demonstrated to be specifically involved in gut response against the parasite *Ceratomyxa shasta* [23] and in gills and skin response against the parasitic ciliate *Ichthyophthirius multifiliis* [26, 27] in rainbow trout and in head kidney response against the ectoparasite *Argulus siamensis* in rohu (*Labeo rohita*) [28]. Moreover, using homozygous isogenic rainbow trout, it was demonstrated that the variable heavy chain domain repertoires of IgT in spleen are induced in spleen after viral infection [29], whereas in the Chines perch (*Siniperca chuatsi*) IgT expression in different tissues was modulated in a different way compared to IgM and IgD after vaccination with *Flavobacterium columnare* [30].

In the present work we identified an IgT from sea bass (*Dicentrarchus labrax* L.) and we characterized this immunoglobulin from a molecular point of view even by analysing in detail its 3D structure and by investigating its basal expression and distribution in mucosal and not-mucosal tissues by real-time PCR and in situ hybridization. The obtained results are of high interest for a view on the possible function of IgT in sea bass especially taking into consideration the involvement of the mucosal tissues in the immune response against pathogens and the high impact and economic value of this species in the Mediterranean aquaculture.

## Methods

### Sea bass IgT cloning

A partial secretory IgT nucleotide sequence was identified after the analysis of a sea bass gills transcriptome [31]. From this sequence two primers were designed (IGTFW 5′-GACCAACTGTGTTTCCTCTGATGC-3′ and IGTRV 5′-GCCATCCTTATAGATGCCAGGGGA-3′) and used in RT-PCR on total RNA extracted with Trisure (Bio-line) solution from gills of a juvenile sea bass (200 g of weight). Leukocytes from sea bass gills were obtained following previously described procedures [32]. RT-PCR was performed using Ready-To-Go RT-PCR Beads (GE Healthcare) and an amplicon of 1034 bp was obtained. The cycling protocol was one cycle of 94 °C for 5 min, 35 cycles of 94 °C for 45 s, 55 °C for 45 s, 72 °C for 45 s, followed by one cycle of 72 °C for 10 min. PCR products (10 μl) were visualised on 1% (w/v) agarose gels containing Gel Red stain (Biotium) and using hyperladder IV (Bioline) as size marker. Controls for the presence of DNA contamination were performed using the RNA samples as template in the PCR cycle. The DNA amplified by PCR was purified using the QIAquick Gel Extraction Kit (QIAgen), inserted into the pGEM-T Easy vector (Promega) and transformed into competent JM109 *Escherichia coli* cells. Plasmid DNA from at least three independent clones was purified using the Wizard Plus SV Minipreps DNA Purification System (Promega) and sequenced using MWG DNA Sequencing Services. The generated sequence was analysed for similarity with other known sequences using the BLAST program [33].

A RACE-PCR using the FirstChoice RLM-RACE kit (Ambion) was performed to obtain the complete secretory IgT sequence. For 5′ RACE, first stand cDNA was synthesized from total gills RNA after adding the 5′ RACE adapter to RNA following the manufacturer’s instruction. A PCR was performed using this cDNA with the IGT1RV1 primer (5′-GAGTGAGTAGACAGGACTGGG-3′), designed on the already known partial IgT sequence, and the 5′ RACE Outer primer of the RLM-RACE kit (5′-GCTGATGGCGATGAATGAACACTG-3′). A 709 bp amplicon containing the 5′ end of the IgT sequence was obtained. For 3′ RACE, first-strand cDNA was obtained using the 3′ RACE adapter following the manufacturer’s instruction. A PCR was performed using this cDNA with the IGT1FW primer (5′-CTCCCTGCCACTCAGTGCG-3′), designed on the already known partial IgT sequence, and the 3′ RACE Outer primer of the kit (5′-GCGAGCACAGAATTAATACGACT-3′). A 701 bp amplicon containing the 5′ end of the IgT sequence was obtained.

The full-length secretory IgT sequence was analysed for the presence of a signal peptide, using SignalP software [34], and of N-glycosylation sites (with the NetNGlyc 1.0 Server). Alignment of the sea bass IgT amino acid sequences to other known molecules from other species and phylogenetic tree with IgT, IgM and IgD sequences were carried out using MEGA 4.1 Software [35].

### Three-dimensional modelling of sea bass IgT

Molecular modelling of sea bass IgT has been performed by comparative modelling technique. The search for template structures has been performed by means of BLAST (http://www.blast.ncbi.lnm.nih.gov) by selecting the Protein Data Bank archive. The results suggested the structure of mouse IgG as a template. The crystallographic structure is deposited with PDB id code 1IGT [36]. The 1IGT structure includes four Ig chains: B chain has been used as template for the modelling of 3D structure of sea bass IgT.

Modelling has been performed by using Modeller 9.12 [37]. The modelling protocol used, as assessed in our lab from previous studies (see [38] as an example), implies firstly the generation of ten models, and, successively, the selection of the best one in terms of structural quality. Stereochemistry analyses and energy evaluations have been obtained by Vadar web server [39] and ProsaWeb [40]. Further structural analyses and visualization have been performed by Discovery Studio (Dassault Systèmes BIOVIA, Discovery Studio Modelling Environment, Release 4.5, San Diego: Dassault Systèmes, 2015).

### Sea bass IgT basal expression analysis

To study the IgT basal expression, four sea bass juveniles were sampled and leucocytes from different tissues [brain, spleen, gut, head kidney (HK), gills, thymus and liver] obtained as described before [32]. Total RNA was isolated from each tissue separately with Trisure (Bioline), suspended in DEPC treated water and used for real-time quantitative PCR without pooling the tissue samples coming from the different fishes. For reverse transcription, the BioScript RNase H minus (Bioline) enzyme was used following the manufacturer’s instruction. The expression level of IgT transcript was determined with a Mx3000PTM real time PCR system (Stratagene) equipped with version 4.1 software and using the Brilliant SYBR Green Q-PCR Master Mix (Agilent Technologies) following the manufacturer’s instructions, with ROX as internal passive reference dye. Specific PCR primers were designed for the amplification of 200 bp products from IgT (IgTFW2 5′-CGGACTTCATTCAGTACCCTG-3′ and IgTRV2 5′-CAACTGTACACATCAGGGCC-3′) and 18S ribosomal RNA (18SFW: 5′-CCAACGAGCTGCTGACC-3′; 18SRV: 5′-CCGTTACCCGTGGTCC-3′), used as house-keeping gene. The primers were selected to amplify a region of the IgT conserved domain that was present in all cloned IgT isoforms. The expression of IgM (Accession Number FN908858) in the same samples was monitored using the primers: IGFW 5′-GAGCTGCAGAAGGACAGTG-3′ and IGRV 5′-TCAGACTGGCCTCACAGCT-3′). 10 ng of cDNA template was used in each PCR reaction. The PCR conditions were 95 °C for 10 min, followed by 35 cycles of 95 °C for 45 s, 52 °C for 45 s and 72 °C for 45 s. Triplicate reactions were performed for each template cDNA and the template was replaced with water in all blank control reactions. The analysis was carried out using the endpoints method option that causes the collection of the fluorescence data at the end of each extension stage of amplification. A relative quantitation has been performed, comparing the levels of the target transcripts (IgT and IgM) to a reference transcript (calibrator, in this case the IgT and IgM expression in the brain, respectively). A normalizer target (18S ribosomal RNA) is included to correct for differences in total cDNA input between samples. The results are expressed as the mean ± SD of the results obtained from the four considered fishes.

### Sea bass IgT expression after infection with nodavirus

The in vivo IgT and IgM expression was investigated on larvae sea bass specimens experimentally infected with viral encephalopathy and retinopathy virus (VERv) or nervous necrosis virus (NNV), a virus belonging to the *Nodaviridae* family, genus *Betanodavirus*, that is one of the most significant viral pathogens for finfish (see [41]). Briefly, 200 sea bass individuals (30–40 g) were injected intramuscularly (i.m.) with PBS, whereas all fish from other tanks (200 + 200 individuals) were injected i.m. with VERv (10^4^ TCID50/ml-1). The virus dose was tested to induce low mortality in sea bass. For gene expression analyses, fish fish/group/time points (0, 24, 48 and 72 h after injection) were sampled and from each individual fish spleen, gills and muscle were removed. RNA extraction, cDNA preparation and real time PCR analysis were performed as described above using as calibrator one muscle from the time 0 control. The results were expressed as the mean ± SD of the results obtained from four fishes and the differences from the control were considered significant if p < 0.05 using the two-way ANOVA analysis following by Bonferroni’s post-test.

### In situ hybridization (ISH) for sea bass IgT

#### Synthesis of RNA probes

Cells from gills, gut and liver of sea bass juveniles (20–30 g) were obtained by tissue teasing and suspended in Tripure (Roche). Total RNA was extracted following manufacturer’s instructions and isolated in DEPC treated water. RT-PCR was performed with Ready-To-Go RT-PCR beads (Amersham) using 1 μg total RNA and 0.5 μg random primers [pd(N)6] in a total 50 μl volume. The primers IgTISHFW (5′-GATGTAGCTGGCACTACCT-3′) and IgTISHRW (5′-TCTCTAGCAGCAGAACAGC-3′) were designed to amplify a 426 bp product corresponding to the constant region of sea bass IgT. Reactions were run using the Mastercycler personal (Eppendorf). The cycling protocol was: 1 cycle of 94 °C for 5 min, 35 cycles of 94 °C for 45 s, 55 °C for 45 s, 72 °C for 45 s, followed by 1 cycle of 72 °C for 10 min. PCR products (15 ml) were visualised on 1% agarose gels containing ethidium bromide (10 ng/ml) using Hyperladder IV (Bioline) as size marker. DNA amplified by PCR was purified using the QIAquick Gel Extraction Kit (QIAgen), inserted into the pGEM-T Easy vector (Promega) and transfected into competent JM109 *E. coli* cells. Plasmid DNA from three independent clones was purified using the Wizard Plus SV Minipreps DNA Purification System (Promega) and sequenced using MWG DNA Sequencing Services. Sequences generated were analysed for similarity with other known sequences using the BLAST program.

The anti-sense probe was obtained using the selected plasmid clone as target in a PCR reaction with primers IgTISHFW and SP6 (5′-GCATTTAGGTGACACTATAGAATAG-3′). The cycling protocol was: 1 cycle of 94 °C for 5 min, 35 cycles of 94 °C for 45 s, 48 °C for 45 s, 72 °C for 45 s, followed by 1 cycle of 72 °C for 10 min. The sense probe (for negative control of ISH) was obtained using the selected plasmid clone as target in a PCR reaction with primers T7 (5′-TAATACGACTCACTATAGGG-3′) and IgTISHRW. The cycling protocol was: 1 cycle of 94 °C for 5 min, 35 cycles of 94 °C for 45 s, 54 °C for 45 s, 72 °C for 45 s, followed by 1 cycle of 72 °C for 10 min. The fragments obtained were purified by Quick Clean Kit (Bioline) and used to synthesize digoxigenin-labeled RNA probes with the DIG-RNA Labeling Kit (Roche) following manufacturer′s instructions.

#### Staining procedures

Dissected gills, gut (anterior, middle and posterior portions) and liver from 1 year-old specimens (N = 20) were fixed overnight at room temperature in 4% paraformaldehyde in 0.01 M, pH 7.4 phosphate-buffered saline (PBS) and gradually dehydrated before paraffin wax embedding. Serial sections (5 μm) were collected on poly-l-lysine coated slides, air-dried overnight at 37 °C and stored at room temperature for subsequent investigation. Sections were dewaxed with xylene, rehydrated in graded ethanol series and DEPC treated water, and then washed with water before proteinase K (Sigma-Aldrich) digestion. The concentration of proteinase K was 1 μg/ml. The digestion was stopped by immersion in cold DEPC water. Acetylation was performed by incubating sections in 0.25% acetic anhydride in 85 mM Tris–HCl buffer containing 0.2% acetic acid and 0.02 M ethylenediaminetetraacetic acid (EDTA) for 10 min. The sections were washed with DEPC water and gradually dehydrated. A dilution profile was performed for the probes with concentrations varying from 0.3 to 0.6 ng/μl and the optimal one resulted 0.45 ng/μl. Following overnight incubation at 45 °C, the sections were washed with 2× saline-sodium citrate (SSC) buffer at room temperature, then with 0.2× SSC at 55 °C for 90 min and incubated in 20 μg/ml RNAaseA in 0.01 M Tris–HCl containing 0.5 M NaCl and 1 mM EDTA for 30 min. Sections were transferred to Buffer 1 (0.1 M Tris containing 0.15 M NaCl and 1% blocking reagent) for 1 h, then to Buffer 2 (0.1 M Tris containing 0.15 M NaCl, 0.5% BSA and 0.3% Triton X-100) for 30 min. The sections were incubated for 2 h at room temperature with alkaline phosphatase-conjugated anti-digoxigenin antibody (Fab fragment; Roche Diagnostic, Germany) diluted 1:1000 in Buffer 2, then washed with 0.1 M Tris containing 0.15 M NaCl and Buffer 3 (0.1 M Tris containing 0.1 M NaCl and 50 mM MgCl_2_). Bound antibody was localized using nitro blue tetrazolium chloride and 5-bromo-4-chloro-3-indolyl-phosphate (Roche Diagnostic, Germany) incubating the sections overnight at room temperature. Thereafter, sections were washed in distilled water and mounted with 50% glycerol. Light microscopy images were captured using a computer-assisted image analysis system which includes a Zeiss microscope equipped with a colour video camera (Axio Cam MRC, Arese, Milano Italy) and a software package (KS 300 and AxioVision).

## Results

### Sea bass IgT cloning

**Fig. 1:**
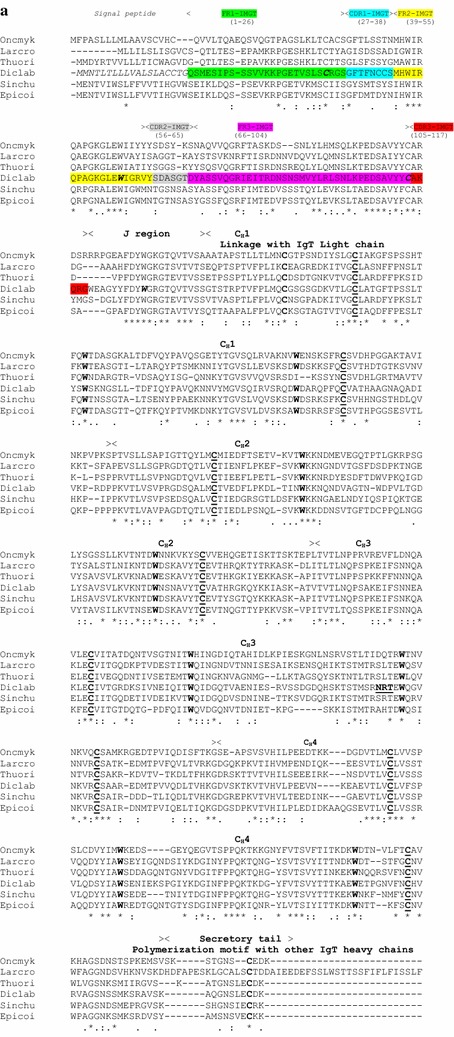
Alignment of the predicted sea bass IgT heavy chain amino acid sequence with other known IgT molecules. The amino acids of the signal peptide in the sea bass sequence are in *italics*. The position of the framework (FR) and CDR regions for the sea bass IgT V-DOMAIN (following the IMGT numbering) is indicated *above the sequences*, together with the J region, the four C_H_ domains and the secretory tail; in this region the typical conserved amino acids in the sea bass sequence are in *bold and italics*. The N-linked glycosylation site in the sea bass IgT sequence is evidenced (see the C_H_3 domain). The conserved amino acids are indicated with an *asterisk* below the sequences, while *dot* and *semicolon* showed amino acids with conserved physical and chemical properties. The conserved cysteine (in *bold and underlined*) and tryptophan (in *bold*) residues that are required for the correct folding of the immunoglobulin superfamily (IgSF) C_H_ domains are highlighted along the sequences. The conserved cysteine residues possibly involved in the disulphide bond with the IgT light chain and the ones related to the possible polymerization with other IgT heavy chains are evidenced in *bold* during the sequences. Accession numbers: *Oncorhynchus mykiss* (rainbow trout) AAW66978; *Larimichthys crocea* (large yellow croaker) XP_010754058; *Thunnus orientalis* (Pacific blue tuna) AHC31432; *Dicentrarchus labrax* (sea bass) KM410929; *Siniperca chuatsi* (mandarin fish) AAY42141; *Epinephelus coioides* (*orange-spotted grouper*) GU182366

**Fig. 2:**
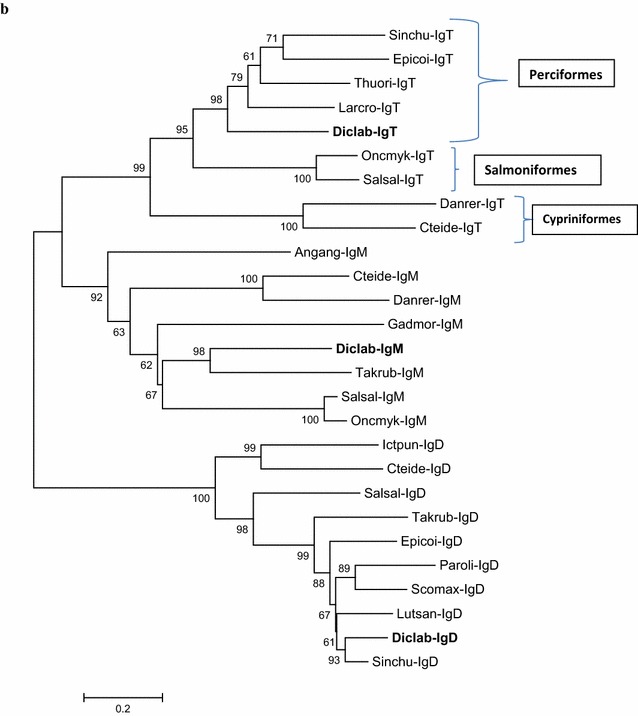
Phylogenetic tree showing the relationship between sea bass IgT, IgM and IgD sequence with other known Ig molecules from teleost fish. The tree was bootstrapped 10,000 times with 50,000 random seeds. The Accession Numbers are the same as indicated in **a**, except for: *Salmo salar* (Atlantic salmon) IgT ACX50292, IgM AAB24064, IgD AF141607; *Danio rerio* (zebrafish) IgT AY643752, IgM AAT67445; *Ctenopharyngodon idella* (grass carp) IgT DQ478943, IgM ABD76396, IgD ADK66818; *Anguilla anguilla* (European eel) IgM ACD76833; *Gadus morhua* (Atlantic cod) IgM CAA41680; *Dicentrarchus labrax* (sea bass) IgM KY173353, IgD KU132360; *Takifugu rubripes* (pufferfish) IgM BAD26619, IgD BAD34542; *Oncorhynchus mykiss* (rainbow trout) IgM AAB27359; *Ictalurus punctatus* IgD (channel catfish) ADF56020; *Epinephelus coioides* (orange-spotted grouper) IgD AFI33218; *Paralichthys olivaceus* (Japanese halibut) IgD BAB41204; *Scophthalmus maximus* (turbot) IgD AFQ38975; *Lutjanus sanguineus* (humphead snapper) IgD AIC33830; *Siniperca chuatsi* (mandarin fish) IgD ACO88906; 0.2 indicates the genetic distance

Several IgT partial sequences were identified from the available gills transcriptome (data not shown) and allowed to design diverse primers for initial cloning and successive 3′- and 5′-RACE amplification of sea bass IgT molecule. The obtained PCR products were used to reconstitute the full-length cDNA corresponding to a sea bass IgT heavy chain subunit (Accession Number KM410929) composed of 552 aa, with a 5′-UTR of 36 bp and a 3′-UTR of 265 bp. The entire coding sequence was successively cloned and sequenced to confirm the correct reconstruction (data not shown). The analysis of the IgT sequence showed the presence of a putative 19 amino acids signal peptide and one potential N-glycosylation site. A multiple alignment of sea bass IgT amino acid sequence with other known IgT teleost sequences was assembled and used to highlight the conservation of the amino acids residues included in the typical Ig structural domains (Fig. 1). With regard to the V-DOMAIN of the IgT sequences the IMGT unique numbering has been used and it gave the possibility to recognize the typical conserved amino acids (cysteine 23, tryptophan 41, cysteine 104 and tryptophan 118) that are always found in this region [42, 43]. The entire C-DOMAIN could be divided in four C_H_ domains as it happens in most IgT heavy chains, with the exception of Fugu [13], that shows two domains (with the lack of C_H_2 and C_H_3), and stickleback [15], with the presence of three domains (with the lack of C_H_2). The four sea bass constant domains contain the cysteine and tryptophan residues required for their correct folding (see Fig. 2) [44] and the additional cysteine found in the C_H_1 domain should be linked to the possibility of a binding with the corresponding IgT light chain. Finally, the secretory tail shows a cysteine residue that could be involved in the process of IgT polymerization [45] that was predicted in rainbow trout [11], but not successively demonstrated [23, 24] in mucus after immunoblot analysis of IgT multimers. IgT, IgM and IgD amino acid sequences from different teleost fish species have been utilized to construct the phylogenetic tree showed in Fig. 2. The three different Ig isotypes are well divided in the tree and in the IgT clade two main branches have been evidenced: one with sequences from Perciformes and Salmoniformes and the other with transcripts from Cypriniformes.

Moreover, we analysed the organization of the sea bass IgT C_H_ exons based on its recently sequenced genome [46]. Despite this chromosome-scale effort was able to place about 85% of the assembled genomic regions to the 24 sea bass chromosomes, about 15% of the genome sequence consists of relatively small contigs which could not be placed to any major scaffold. Curiously, all the IgT C_H_ exons we identified in sea bass are confined to this portion of the genome, suggesting that they may be present in multiple, nearly identical copies, or surrounded by highly repetitive DNA regions which makes quite complicated the assembly of these genomic regions in larger scaffolds. As a matter of fact, we could identify at least five different genomic contigs bearing exons unequivocally pertaining to IgT (C_H_2, C_H_3 and C_H_4), and none of these contigs corresponded to a complete IgH locus. Due to the high apparent fragmentation of IgH loci in the sea bass genome, it remains impossible to establish, based on the data currently available, whether these assembled genomic sequences correspond to five distinct IgT genes and how many IgH loci are actually present in sea bass. The presence of multiple IgT genes, organized in different IgH loci, has been previously reported in other teleosts fish, including salmon (8 genes in two distinct loci) [20] and even in Eupercaria, such as, stickleback (4 genes in four distinct loci) [15]. The complete cloned sequence of the sea bass IgT corresponds almost perfectly to one of the identified IgT genes, with the exception of a few SNPs located at the 3′ of the coding sequence.

### Three-dimensional modelling of sea bass IgT

**Fig. 3:**
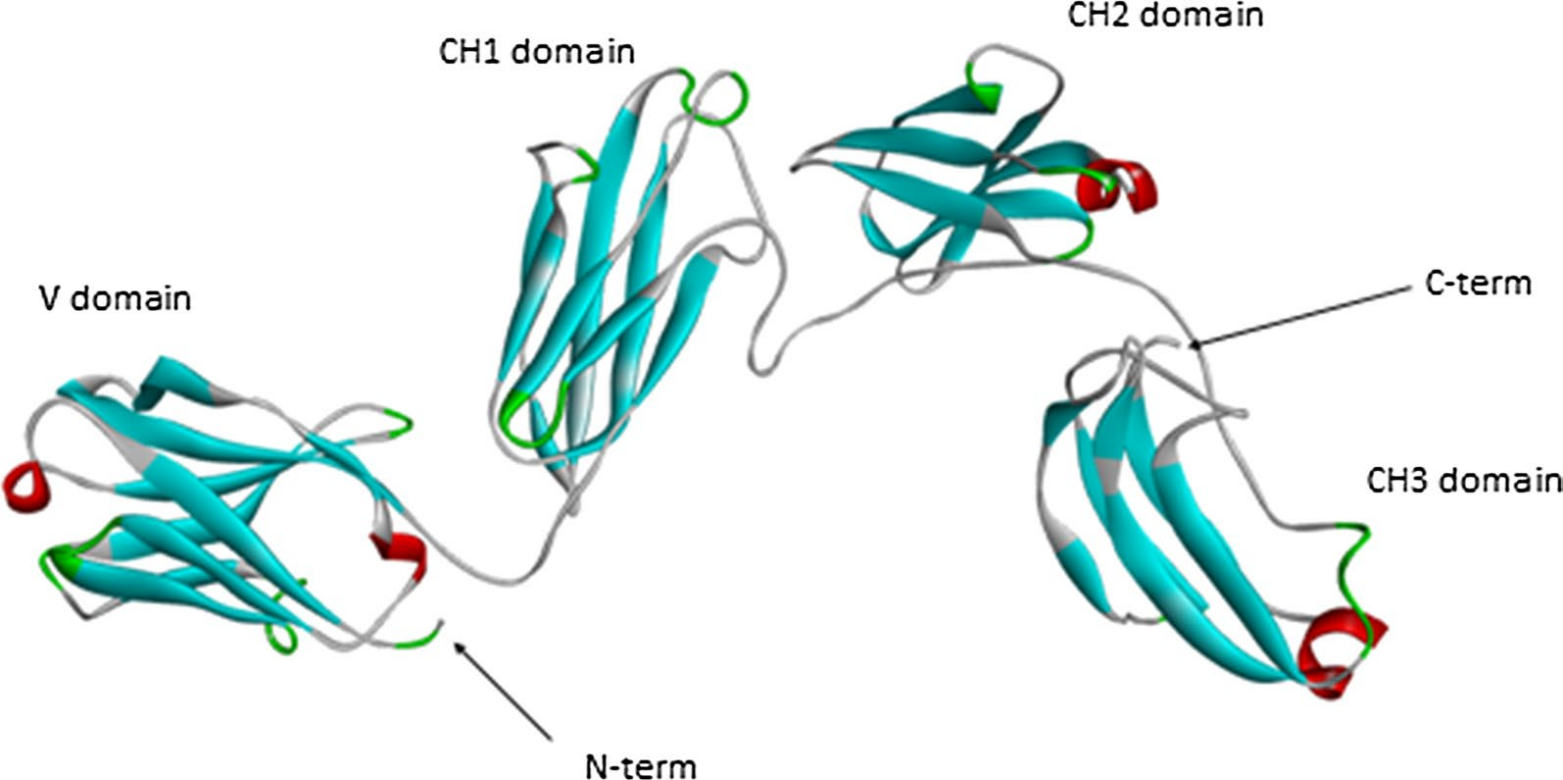
Schematization of the three-dimensional model of sea bass IgT. N- and C-terminus of the model are indicated by *arrows*. The four structural domains are indicated, from *left to right*, as VH, C_H_1-gamma, C_H_2-gamma, and C_H_3-gamma, respectively. Short helices are coloured in *red*, while beta strand are in *cyan*, and turns in *green*

The 3D model of sea bass IgT has been generated by comparative modelling after selection of the best template available in PDB. The template structure selected is identified by PDB code 1IGT, an intact Ig gamma-2A monoclonal antibody from mouse. The structure includes two light chains of 214 residues, named A and C, and two heavy chains of 444 residues, named B and D. The chain used to model the sea bass structure is the B one, and the region of sea bass IgT modelled covers the amino acid region from Lys32 to Met423 (therefore not the entire IgT molecule). This region, aligned to the mouse template, shares 31% of sequence identity and 48% of similarity (i.e., identical or similar residues aligned). The obtained model is shown in Fig. 3. Checks for stereo chemical quality and energy profiles of the model indicate that the sequence is well suited in the 3D structure. The architectural organization resembles that of the template, so we can recognize the immunoglobulin-like architecture, and the presence of the heavy chain domains: variable domain, VH (from N-terminus to residue Gly142); constant domain 1, C_H_1 (from Thr143 to Pro243); constant domain 2, C_H_2 (from Pro244 to Val335); constant domain 3, C_H_3 (from Thr336 to C-terminus of the model). Sea bass IgT contains 11 Cys residues, and the model shows that 4 S–S bridges are possible, i.e. Cys10–Cys84, Cys135–Cys192, Cys229–Cys285, Cys325–Cys385. These residues are also present and form S–S bridges in the mouse IgG template. However, some difference concerns the position of other Cys residues that in mouse IgG are involved in inter-chain bonds, and this aspect will described under “Discussion”.

### IgT basal expression analysis

**Fig. 4:**
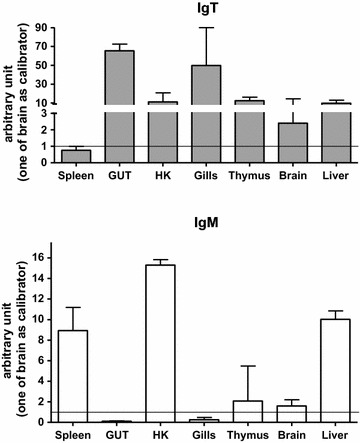
IgT and IgM sea bass basal expression in different tissues. IgT and IgM mRNA levels were expressed as a ratio relative to rRNA 18S levels in the same tissue after real-time PCR analysis using the brain as calibrator. Data were expressed as the mean ± SD

Un-stimulated sea bass juveniles were used to investigate IgT and IgM transcript levels in different organs and tissues (see Fig. 4). The highest IgT expression was found in gut followed by gills, whereas IgM maximum expression was in head kidney, followed by spleen and liver.

### Sea bass IgT expression after infection with nodavirus

**Fig. 5:**
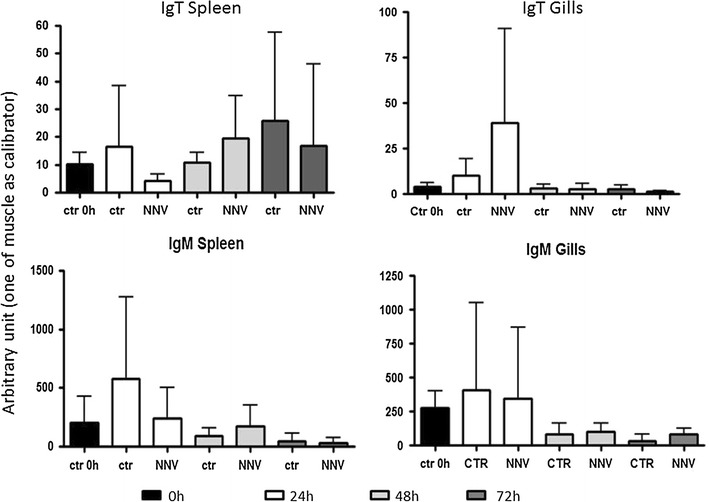
IgT expression after infection with nodavirus. Sea bass IgT and IgM mRNA levels were expressed as a ratio relative to rRNA 18S in the same samples after real-time PCR analysis of spleen and gills leukocytes from four fish infected i.m. with nodavirus and normalized against one muscle from a time 0 control (not showed)

Sea bass is highly susceptible to nodavirus (NNV) in all stages of his life, but larval and juveniles are commonly most affected, reaching mortalities up to 100%. For this reason, we analyzed the IgT expression in sea bass larvae after few days from infection with nodavirus in leukocytes from spleen and gills (see Fig. 5), to investigate the early modulation of this gene due to the immune response. In spleen, IgT expression slightly increased 48 h after the infection and successively decreased at 72 h, being the observed modulations of expression non statistically significant. However, taking into consideration the responses of the single fishes, two of the four tested animals showed a significant IgT increase at 48 h. In gills, a high increase of IgT expression was found 24 h after the infection, followed by a large decrease after 48 h, but still the data were statistically significant only in two single fishes out of the four examined. Moreover, IgM expression in spleen showed only a not statistically significant slight increase at 48 h, whereas in gills no differences were found in the IgM expression at the considered time points.

### In situ hybridization (ISH) for sea bass IgT

**Fig. 6:**
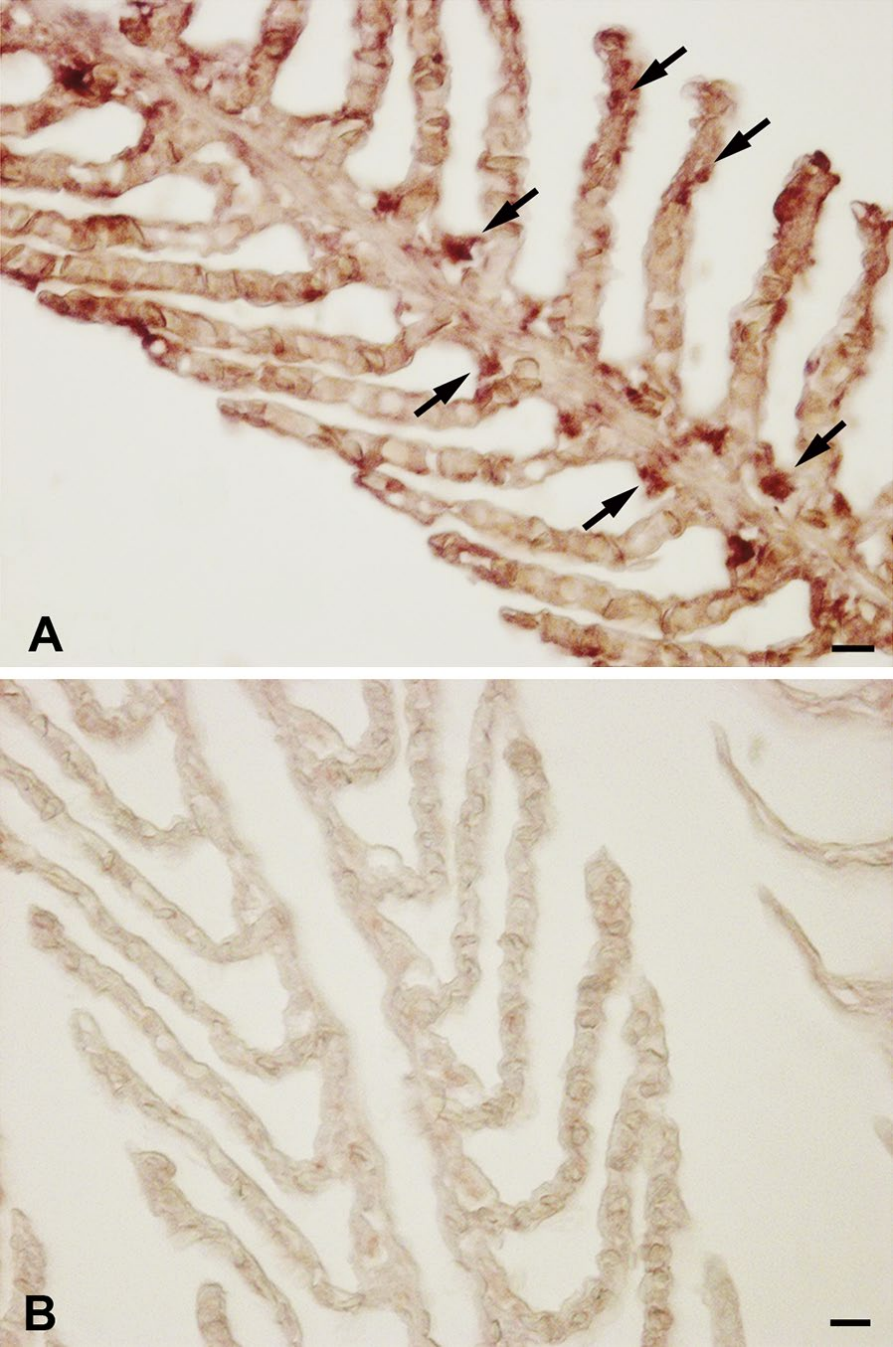
IgT-expressing cells in sea bass gills. **A** IgT expressing cells in sea bass gill filaments and parallel lamellae (*arrows*). **B** Negative control with sense probe. *Scale bars* **A**, **B** 10 µm

**Fig. 7:**
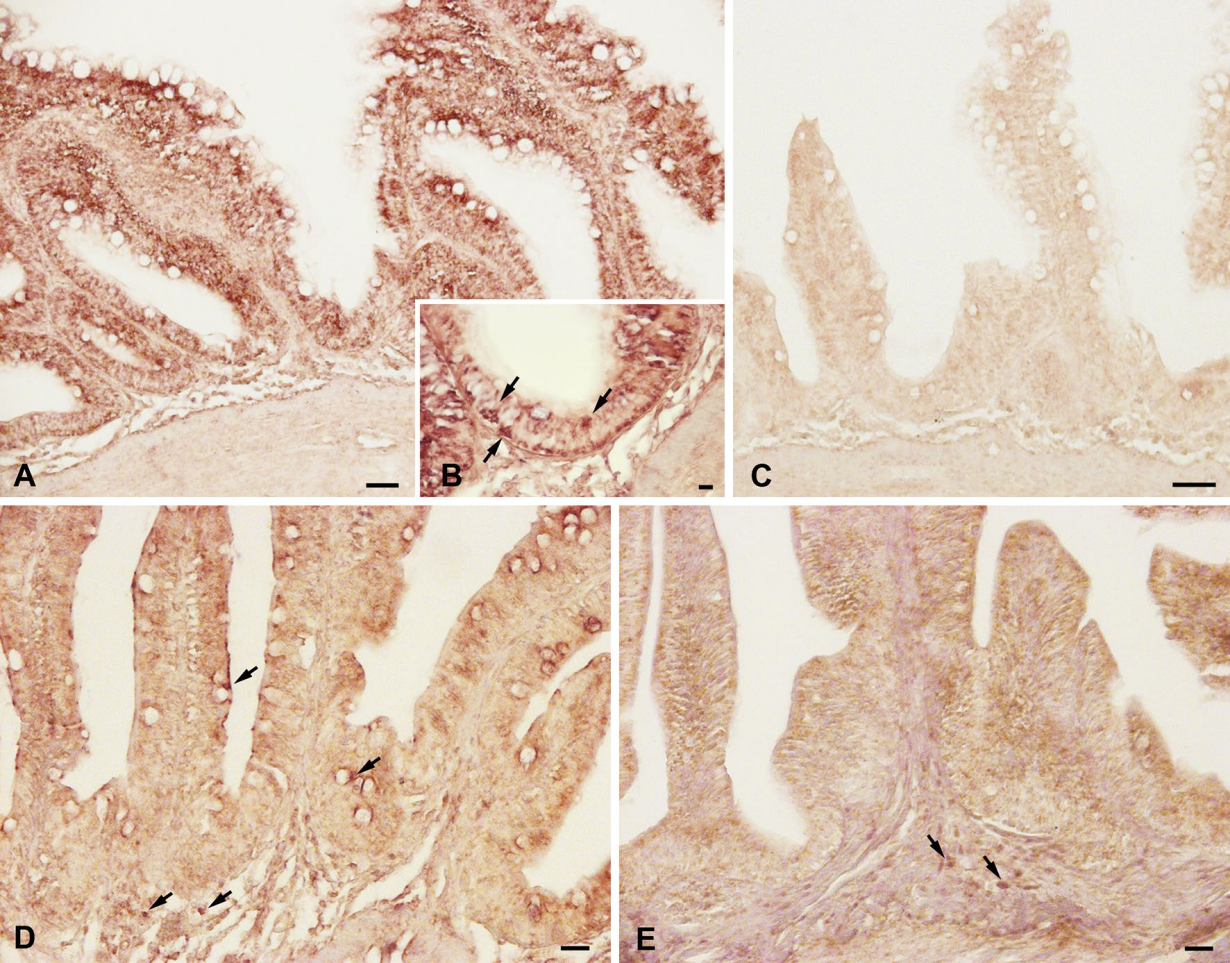
IgT-expressing cells in sea bass posterior, middle and anterior intestine. **A** IgT^+^ cells in the epithelium and lamina propria of posterior segment. **B** Higher magnification showing intraepithelial lymphocytes expressing IgT transcripts. **C** Negative control of posterior intestine with sense probe. **D** IgT^+^ cells in epithelium and lamina propria of the middle segment. **E** Anterior segment showing IgT-expressing cells localized in the lamina propria. *Scale bars* **A**, **D**, **E** 20 µm; **B** 10 µm; **C** 50 µm

**Fig. 8:**
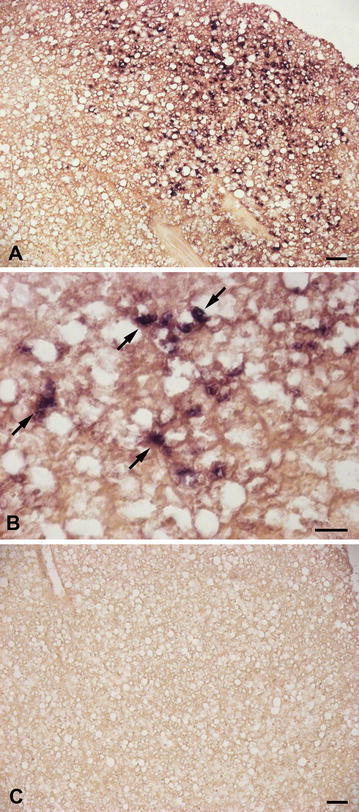
IgT transcripts in the liver. **A** IgT anti sense probe stains positive cells in the hepatic parenchyma. **B** Higher magnification of IgT^+^ cells. **C** No signal was revealed using m-RNA sense probe. *Scale bars* **A**, **B** 20 µm; **C** 50 µm

ISH analysis of gills and intestine cross sections first revealed the presence of IgT-expressing cells in sea bass mucosal tissues (Fig. 6A). In particular, IgT positive cells, with strong positive signals, were distributed in the gill filaments and parallel lamellae located perpendicularly on the filaments. The IgT m-RNA probe recognized several cells localized in the epithelium of the filaments, while rare transcripts were found in the epithelium of the branchial lamellae (Fig. 6A). The use of IgT sense probes did not result in any staining (Fig. 6B). IgT expression was also detected in the different intestinal segments (Fig. 7a–e), although the distribution of IgT expressing cells was regionalized, with a major recruitment in the posterior intestine. In this latter segment, IgT expressing cells were primarily detected both in the epithelium, as intraepithelial basolateral lymphocytes, and in the lamina propria (Fig. 7A, B). No staining was revealed using sense probes (Fig. 7C). In the middle intestine IgT transcripts were identified in some intraepithelial lymphocytes and in isolated cells located in the lamina propria (Fig. 7D), while in the anterior segment rare cells were located in the lamina propria (Fig. 7E). In addition, through ISH, the presence of resident IgT expressing cells was first localized in sea bass liver (Fig. 8A–C). In particular, the IgT mRNA probe allowed identifying scattered IgT positive cells, showing a strong positive signal in the hepatic parenchyma (Fig. 8A, B). It needs to be noticed that IgT expressing cells were mainly dispersed along the outer zone of the organ. No signal was revealed using m-RNA sense probe (Fig. 8C).

## Discussion

In this paper we described the identification of an IgT heavy chain from sea bass (*D. labrax* L.) and we performed its molecular characterization, its expression analysis in different un-stimulated organs and tissues and after in vivo infection with nodavirus and, finally, its localization by in situ hybridisation to preliminary investigate the functional activities of this molecule.

The complete sequence of the secreted IgT heavy chain, obtained from gills cDNA, is composed of 552 aa and its conserved region is constituted of four domains as it happens in all Teleost sequences identified until now except for Fugu [13] and stickleback [15]. In all sequences the C_H_1, which shows high similarity with the C_H_1 region of IgM, and the C_H_4 are conserved; this aspect may be related to their possible role in binding to Ig light chains (C_H_1) or to other Ig heavy chains (C_H_4) through inter-chain disulphide bonds [24]. In the recent identified IgT from an Antarctic fish, the *Trematomus bernacchii*, we have a peculiar situation with the lack of most of the second constant domain that maintained only few amino acid residues [19]. The performed phylogenetic analysis (Fig. 2) showed that sea bass IgT sequence clustered with IgT sequence from other Perciformes, whereas the sequences from Cypriniformes resulted quite different from the other teleost IgT and were located in a different clade.

The 3D structure of sea bass IgT has been generated with the template structure of a mouse Ig, which results best template available in PDB. Intra-chain S–S bonds are similarly located in the four domains, while differences are found between the two sequences for the position of other cysteines not involved in intra-chain S–S bonds. In mouse Ig gamma, Cys128 forms an inter-chain S–S bond with Cys214 of the light chain (A chain in the PDB file). In similar position, sea bass IgT has Cys123 in the C_H_1 domain, not involved in intra-chain bonds that could be suitable for an inter-chain bond. Cys19 and Cys20 residues in sea bass IgT do not appear able to form intra-chain bonds, and they have no corresponding Cys residue in the mouse sequence. On the contrary, mouse Ig gamma has three Cys residues in position 237, 240, and 242, not involved in intra-chain bonds, for which there is no corresponding Cys residue in sea bass IgT. These residues in mouse IgG form inter-chain S–S bonds with the corresponding residue (i.e., Cys237 of B chain linked to Cys237 of D chain) of another Ig gamma heavy chain in the crystallographic structure. The modelling of the multimeric structure of sea bass IgT was not possible in the absence of the light chain sequence; therefore, for the same reason, it was not possible to more deeply check the suitability of the model to form inter-chain S–S bonds.

IgT/IgZ ontogeny has been studied in zebrafish, trout and Fugu and it seemed evident that transcripts related to these molecules are expressed from the very early developmental stages (as an example 4 days post-fertilization in Fugu and 22 days in rainbow trout) [11, 13] . In zebrafish two IgZ isoforms were detected which showed different expression times: IgZ1 was detected few days post-fertilization, whereas IgZ2 only 14 days post-fertilization [47]. In sea bass we analysed the basal expression of IgT and IgM in different organs and tissues and we found that gut and gills, important mucosal organs, showed high IgT transcripts levels and this was for us the first evidence of the possible involvement of sea bass IgT in mucosal immune responses as already demonstrated in rainbow trout [23]. Moreover, we have observed expression, but at a lesser extent, in other important organs linked to the immune responses, like thymus, head kidney and liver. IgM levels, on the contrary, were high in head kidney, spleen and liver, organs that are involved in systemic immune responses. In general, however, the basal expression seems an aspect peculiar in relation to the investigated fish species. We have examples, like zebrafish, where IgT transcripts are found only in primary lymphoid organs (head kidney and thymus) in adult individuals, while IgM expression is widespread in both primary and secondary lymphoid organs [12]. On the other hand in other species, such as trout, fugu and Atlantic salmon the two expression patterns are very similar [11, 13, 17]. Moreover, in carp IgT is expressed even in non-lymphoid organs [48] and, after specific infection with fish parasites, in carp and trout IgT becomes highly over-expressed in mucosal organs (gut and gills) [23, 48]. Sea bass IgT expression was modulated, even if the variations were not statistically significant although not considering the response of single fishes, after i.m. infection with nodavirus both in spleen and gills. The IgT increase was more evident in gills, an important mucosal tissue, and less in spleen. The involvement of sea bass IgT in the immune response against bacterial [49] and virus [50] pathogens has been previous evidenced in gut, but this is the first time of a similar investigation in gills and spleen. It has to be noted that in rainbow trout a robust clonal response involving few variable heavy chain domain repertories has been evidenced in spleen after challenge with a rhabdovirus [29] and that it has been demonstrated that in rainbow trout, after pathogen exposure, IgT^+^ B cells proliferate and generate specific IgT in the gills and in the skin [27, 51].

However, while extensive studies have been performed on the distribution of IgM-producing cells in lymphoid tissues [52], little information is available on the distribution of IgT/IgZ producing cells in fish species. To this purpose we performed in situ hybridization to detect IgT-producing cells in sea bass mucosal tissues, gills and gut. IgT transcripts were localized in gill filaments and parallel lamellae showing strong positive signals, while in Fugu a strong expression of IgZ was seen in the gill epithelial cells [13] and in mandarin fish a moderate expression of IgZ positive cells was detected loosely located along gill filaments [30], with a similar distribution to that of IgM transcripts. Functional studies in mandarin fish, that measured the levels of specific transcripts for IgM, IgD and IgZ in mucosal secretions after vaccination with the inactivated bacteria *Flavobacterium columnare*, demonstrated an increase of Igs in gills, although with different kinetics for the three Ig isotypes, suggesting that in the gill associated lymphoid tissue B cells could play important roles in protecting fish from pathogens [30]. In accordance with this, data obtained in sea bass showed that antibody production was early induced in the gills of juveniles after immersion in *Photobacterium damselae* ssp. piscicida bacterin demonstrating that the gill is a major organ for antibody secreting cell production [53].

In situ hybridization with a IgT-mRNA probe confirmed the presence of IgT transcripts also in the sea bass gut. To notice, sea bass IgT expression was regionalized with a major expression in the posterior intestine, data that points to the hindgut as the segment in which the recruitment of IgT^+^ cells is most significant in this species. Also in rainbow trout gut IgT^+^ cells distribution was regionalized, although in this species the pyloric caeca was the area with a major recruitment of B cells in response to oral vaccination, as demonstrated through both real time PCR and immune-histochemistry [54]. In sea bass IgT^+^ cells were mostly intra-epithelial leukocytes (IELs) in anterior and middle segments, while they were localized in the lamina propria (LP) in all segments. However, the location of IgT-producing cells is not coincident among different fish species. In fact, although in rainbow trout IgT^+^ cells were mostly IELs in all segments and rarely they were localized in the LP in the midgut and hindgut regions [54], after vaccination, the cells found in the LP can infiltrate the epithelium [54]. Interestingly, in mandarin fish no IgZ positive cells could be detected in intestine, while numerous IgM positive cells were located abundantly in both submucosa and lamina propria of posterior intestine and sometimes nearing the goblet cells. In sea bass, IgM^+^ cells were more common in the epithelium than in lamina propria and T cells were mainly found in the posterior intestine [55]. The presence of T cells and IgM ^+^ and IgT ^+^ cells in the mucosa of the sea bass posterior intestine seems one of the factors that could account for its capacity to respond to immune stimuli.

Overall, numerous observations, recently reviewed by Parra et al. [25], indicated that, similarly to IgA and IgM in mammals, the transportation of polymeric IgT and IgM in teleost fish is mediated by pIgR. However, as demonstrated in mouse, rat and rabbit, only a small proportion of IgA synthesized in the lamina propria of intestinal mucosa enters the gut lumen directly. The majority goes through the mesenteric lymph, then in the bloodstream, and is finally transported into bile by hepatocytes to protect the intestinal epithelium. Finally, in sea bass IgT transcripts were also found in the liver. In particular, the IgT mRNA probe allowed identifying scattered IgT positive cells, showing a strong positive signal, in the hepatic parenchyma, providing clear evidence in support of the existence of a resident immune cell population in this tissue. The result validates previous data obtained in trout that provided evidence for the existence of an intrahepatic immune cells population which is 15–29% of the non-hepatocyte cells in the liver [56]. However, the present study, to the best of our knowledge, demonstrates for the first time the existence of an intraparenchymal lymphocyte IgT population in sea bass liver. In rainbow trout it has been evidenced the presence of IgT^+^ cells and the liver immune response after infection with the viral hemorrhagic septicemia virus has been assessed [57].

## Conclusions

From this analysis we could conclude that sea bass IgT presents different molecular aspects in agreement with previously identified homologs from other Teleost fish species and that expression and localization data strongly suggest its possible involvement in mucosal immune responses against pathogens. Much work needs to be addressed to study more in detail this last aspect, for the importance of mucosal antibodies as a target to study the efficacy of oral vaccines (via feed) that are used in fish immersion vaccination, an ideal method for the aquaculture sector [58, 59], and to investigate the importance of the IgT population evidenced in the sea bass liver.
